# Supplementary material for: Combating errors in quantum communication: an integrated approach
Source: Sci Rep. 2023 Feb 20;13:2979. doi: 10.1038/s41598-023-30178-x (PMC9941483; doi:10.1038/s41598-023-30178-x)
Supplement: Supplementary file 1 — Supplementary Information 1. [file 41598_2023_30178_MOESM1_ESM.pdf]

Combating errors in quantum communication: an integrated approach: supplemental document

RAJNI BALA[1,*], SOORYANSH ASTHANA[1], AND V. RAVISHANKAR[1]

[1] Department of Physics, Indian Institute of Technology Delhi, New Delhi, 110016, India.
[*] Rajni.Bala@physics.iitd.ac.in

1. PROOF OF NONEXISTENCE OF INVARIANTS BELONGING TO THE SECOND FAMILY IN A GENERALISED PAULI CHANNEL

Consider an operator $O \equiv \sum_{m,n} c_{mn} X^m Z^n$, which evolves in a generalised channel in the following manner,

$$O \to O' = \sum_{r,s} p_{rs} (X^r Z^s)^\dagger O (X^r Z^s). \tag{S1}$$

Employing the relation $ZX = \omega XZ$, the above equation assumes the following form,

$$O' = \sum_{r,s} p_{rs} \sum_{m,n} \omega^{nr-sm} c_{mn} X^m Z^n. \tag{S2}$$

The above equation indicates that for arbitrary values of p_{rs}, there cannot exist two operators that either scale by the same factor say, λ or scale such that $\lambda_2 = \lambda_1^r$, where $\lambda_{1,2}$ represents the scaling with which the expectation values of the two operators is changed. That is why there does not exist any invariant from the second or the third family for a generalised Pauli channel.

2. ENUMERATION OF INVARIANTS IN A GENERALISED FLIP CHANNEL

The operators appearing in the two sets of invariants are unitary and hence, each quantity gives two real invariants. Therefore, to ensure the independence in the information provided by invariants, the inverse operators should be excluded. Thus, the ranges of m and l are to be decided accordingly. We do this for odd and even N separately.

Odd N

Range for the first family of invariants: $m \in \{1, 2, \cdots, \frac{N-1}{2}\}$.
Range for the second family: $m \in \{1, 2, \cdots, \frac{N-1}{2}\}$, and $l \in \{1, 2, \cdots, N-1\}$.

Even N

Range for the first family: $m \in \{1, 2, \cdots, \frac{N}{2}\}$.
Range for the second family: For $m \in \{1, 2, \cdots, \frac{N}{2} - 1\}$, $l \in \{1, 2, \cdots, N-1\}$ and whenever $m = \frac{N}{2}$, $l \in \{1, 2, \cdots, \frac{N}{2}\}$.
The difference in the range of m and l for even and odd N arises due to the fact that for even N, there is an operator, $U^{\frac{N}{2}}$, which is inverse of itself and therefore, has only one independent invariant.

Given the range of m and l for both even and odd N, it is clear that the set $\mathcal{I}_1^{(m)}$ provides $(N-1)$ independent quantities whereas the set $\mathcal{I}_2^{(ml)}$ provides $(N-1)^2$ independent quantities which is quadratic in N. In all, there are $(N^2 - N)$ independent invariants.

3. INVARIANTS FOR A QUBIT

In this section, we have considered various noisy channels for a qubit, which even as of today, are predominantly used for communication protocols such as quantum key distribution, quantum

teleportation, quantum secret sharing. In particular, we have considered a bit-flip channel, a phase-flip channel, a depolarization channel, and an amplitude–damping channel, and have identified invariants for the same. These noisy channels are significant not merely from theoretical considerations, but also from an experimental viewpoint. The effect of these noisy channels on various communication protocols such as quantum teleportation, superdense coding, quantum state sharing has been studied[1–5].

We first recall the set of Kraus operators for each of the noisy channel considered here.

- Bit flip channel: $E_1 = \sqrt{1-p}\,\mathbb{1},\ E_2 = \sqrt{p}\,\sigma_x$.
- Phase flip channel: $E_1 = \sqrt{1-p}\,\mathbb{1}, E_2 = \sqrt{p}\,\sigma_z$.
- Combined bit and phase flip: $E_1 = \sqrt{1-p}\,\mathbb{1},\ E_2 = \sqrt{p}\,\sigma_y$.
- Depolarising channel: $E_1 = \sqrt{1-\frac{3p}{4}}\mathbb{1},\ E_2 = \sqrt{\frac{p}{4}}\sigma_x,\ E_3 = \sqrt{\frac{p}{4}}\sigma_y,\ E_4 = \sqrt{\frac{p}{4}}\sigma_z$.
- Amplitude damping channel:

$$E_0 = \frac{1}{2}\Big((1+\sqrt{1-q})\mathbb{1} + (1-\sqrt{1-q})\sigma_z\Big), \quad E_1 = \sqrt{q}\frac{\sigma_+}{2}; \quad q \in [0,1].$$

- Generalised amplitude damping channel:

$$K_1 = \frac{\sqrt{p_1}}{2}\Big((1+\sqrt{1-q})\mathbb{1} + (1-\sqrt{1-q})\sigma_z\Big), \quad K_2 = \frac{\sqrt{p_1 q}}{2}\sigma_+;$$
$$K_3 = \frac{\sqrt{p_2}}{2}\Big((1+\sqrt{1-q})\mathbb{1} - (1-\sqrt{1-q})\sigma_z\Big), \quad K_4 = \frac{\sqrt{p_2 q}}{2}\sigma_-, \text{ where } q \in [0,1], \text{ and } p_1 + p_2 = 1.$$

The invariants for all these noisy channels are identified in the table (S1).

**Noisy channel**	**First family of invariants**	**Second and third families of invariants**
Bit-flip channel	$\langle\sigma_x\rangle$	$\frac{\langle\sigma_y\rangle}{\langle\sigma_z\rangle}$
Phase-flip channel	$\langle\sigma_z\rangle$	$\frac{\langle\sigma_x\rangle}{\langle\sigma_y\rangle}$
Combination of bit and phase-flip channel	$\langle\sigma_y\rangle$	$\frac{\langle\sigma_x\rangle}{\langle\sigma_z\rangle}$
Depolarizing channel	–	$\frac{\langle\sigma_x\rangle}{\langle\sigma_z\rangle}, \frac{\langle\sigma_y\rangle}{\langle\sigma_z\rangle}$
Amplitude damping channel	–	$\frac{\langle\sigma_x\rangle}{\langle\sigma_y\rangle}, \frac{\langle\sigma_x\rangle\langle\sigma_y\rangle}{\langle\pi_z^-\rangle}$
Generalised amplitude damping channel	–	$\frac{\langle\sigma_x\rangle}{\langle\sigma_z\rangle}$

Table S1. Sets of invariants for various noisy channels of a qubit.

This concludes our discussion of invariants for a qubit passing through different noisy channels.

4. SECURITY OF THE PROPOSED QKD PROTOCOL

In this section, we examine the security of the protocol proposed in section "Application" of the manuscript against intercept-resend and entangle-and-measure attacks.

Intercept-resend attack: Here, Eve intercepts and sends the post-measurement state to Bob. But, then, her presence will be given away with decoy states, which we recall, are sent randomly in some of the rounds. Since Eve cannot differentiate between the decoy states and the states that are employed to transfer information, she ends up measuring the decoy states. As a result of Eve's measurement, the state of system may change, which will be reflected in the sifting process when Bob reveals his measurement observables and the corresponding outcomes. Thus, the presence of Eve will be detected.

Entangle-and-Measure attack: Here, Eve entangles her ancillary system with the state travelling to Bob. By performing a measurement on her system, she retrieves information on Bob's state. However if she does so, the statistics of the decoy states will change.

In this way, the proposed protocol is secure against these attacks. A more general security analysis for the proposed protocols constitute a separate study and will be taken up elsewhere.

5. REMOTE TRANSFER OF INFORMATION THROUGH A DEPOLARISING CHANNEL

Remote transfer of information requires sharing of entangled states between two parties. However, to share such states, at least one of the subsystem has to travel through channels, which are necessarily noisy. As a consequence, the state of the entire system changes. Given this situation, a good question to ask is whether information can be transferred in an error-immune manner. Again, the answer is in the affirmative if we employ the information encoding scheme proposed in the formalism of the manuscript. That is, if it is encoded in invariants for a noisy channel, it can be transferred in an error-immune manner. As a prototype, consider a maximally entangled two-qubit state with one of the qubits passing through a depolarising channel.

Let Alice possess a singlet state,

$$\rho = \frac{1}{4}\big(\mathbb{1} - \vec{\sigma}_1 \cdot \vec{\sigma}_2\big). \tag{S3}$$

She sends one of the two qubits to Bob. Because the channel is depolarising, the final state shared by Alice and Bob, which is corrupted, will have the form,

$$\rho' \equiv p_0\rho + p\big(\sigma_{2x}\rho\sigma_{2x} + \sigma_{2y}\rho\sigma_{2y} + \sigma_{2z}\rho\sigma_{2z}\big) = \frac{1}{4}\big(\mathbb{1} - \alpha\vec{\sigma}_1 \cdot \vec{\sigma}_2\big), \tag{S4}$$

where $\alpha = (1-4p)$, $0 \leq p_0, \; p \leq 1$ and $p_0 + 3p = 1$. Alice wants to transmit information of her measurement to Bob without any errors. Due to the transmission of the second qubit through the noisy channel, she cannot send full information about her measurement direction. However, she can send, if not all, some partial error–free information about her measurement. This can be done by encoding information about her measurement in the invariants of a depolarising channel. This can be seen as follows:

Alice performs a measurement of an observable $\vec{\sigma}_2 \cdot \hat{m}$ on the qubit in her possession. As a result of this measurement, the state of Bob will be, $\rho_B = \frac{1}{2}\big(\mathbb{1} \pm \alpha\vec{\sigma}_2 \cdot \vec{m}\big)$. Bob can retrieve information encoded in the invariants,

$$I_1 = \frac{\langle\sigma_{2y}\rangle}{\langle\sigma_{2x}\rangle} = \frac{m_y}{m_x}, \quad I_2 = \frac{\langle\sigma_{2y}\rangle}{\langle\sigma_{2z}\rangle} = \frac{m_y}{m_z}, \tag{S5}$$

since these are independent of α. These invariants provide an error-free information about Alice's measurement. In this way, Alice is able to remotely transfer information about her measurement to Bob in an error-immune manner. The performance of the protocol is limited by sensitivity of the detector.

REFERENCES

1. R. Fortes and G. Rigolin, "Fighting noise with noise in realistic quantum teleportation," Phys. Rev. A **92**, 012338 (2015).
2. R. Fortes and G. Rigolin, "Probabilistic quantum teleportation in the presence of noise," Phys. Rev. A **93**, 062330 (2016).

3. Z. Shadman, H. Kampermann, C. Macchiavello, and D. Bruss, “Optimal super dense coding over noisy quantum channels,” New J. Phys. **12**, 073042 (2010).
4. Y.-L. Li, D.-M. Wei, C.-J. Zu, and X. Xiao, “Enhanced superdense coding over correlated amplitude damping channel,” Entropy **21**, 598 (2019).
5. M.-M. Wang, W. Wang, J.-G. Chen, and A. Farouk, “Secret sharing of a known arbitrary quantum state with noisy environment,” Quantum Inf. Process. **14**, 4211–4224 (2015).
